# Behavioral Phenotyping of Parkin-Deficient Mice: Looking for Early Preclinical Features of Parkinson's Disease

**DOI:** 10.1371/journal.pone.0114216

**Published:** 2014-12-08

**Authors:** Daniel Rial, Adalberto A. Castro, Nuno Machado, Pedro Garção, Francisco Q. Gonçalves, Henrique B. Silva, Ângelo R. Tomé, Attila Köfalvi, Olga Corti, Rita Raisman-Vozari, Rodrigo A. Cunha, Rui D. Prediger

**Affiliations:** 1 Departamento de Farmacologia, Centro de Ciências Biológicas, Universidade Federal de Santa Catarina, UFSC, Florianópolis, 88049-900, SC, Brazil; 2 CNC - Center for Neuroscience and Cell Biology, University of Coimbra, 3004-517, Coimbra, Portugal; 3 CNRS UMR 7225, Hôpital de la Salpêtrière—Bâtiment, ICM (Centre de Recherche de l'Institut du Cerveau et de la Moelle épinière), CRICM, Thérapeutique Expérimentale de la Neurodégénérescence, Université Pierre et Marie Curie, UPMC, 75651, Paris, France; 4 Department of Life Sciences, Faculty of Sciences and Technology, University of Coimbra, 3000-456, Coimbra, Portugal; 5 Faculty of Medicine, University of Coimbra, 3005-504, Coimbra, Portugal; Federal University of Rio Grande do Norte, Brazil

## Abstract

There is considerable evidence showing that the neurodegenerative processes that lead to sporadic Parkinson's disease (PD) begin many years before the appearance of the characteristic motor symptoms. Neuropsychiatric, sensorial and cognitive deficits are recognized as early non-motor manifestations of PD, and are not attenuated by the current anti-parkinsonian therapy. Although loss-of-function mutations in the *parkin* gene cause early-onset familial PD, Parkin-deficient mice do not display spontaneous degeneration of the nigrostriatal pathway or enhanced vulnerability to dopaminergic neurotoxins such as 6-OHDA and MPTP. Here, we employed adult homozygous C57BL/6 mice with *parkin* gene deletion on exon 3 (*parkin* ^−/−^) to further investigate the relevance of Parkin in the regulation of non-motor features, namely olfactory, emotional, cognitive and hippocampal synaptic plasticity. *Parkin* ^−/−^ mice displayed normal performance on behavioral tests evaluating olfaction (olfactory discrimination), anxiety (elevated plus-maze), depressive-like behavior (forced swimming and tail suspension) and motor function (rotarod, grasping strength and pole). However, *parkin* ^−/−^ mice displayed a poor performance in the open field habituation, object location and modified Y-maze tasks suggestive of procedural and short-term spatial memory deficits. These behavioral impairments were accompanied by impaired hippocampal long-term potentiation (LTP). These findings indicate that the genetic deletion of *parkin* causes deficiencies in hippocampal synaptic plasticity, resulting in memory deficits with no major olfactory, emotional or motor impairments. Therefore, *parkin* ^−/−^ mice may represent a promising animal model to study the early stages of PD and for testing new therapeutic strategies to restore learning and memory and synaptic plasticity impairments in PD.

## Introduction

Parkinson's disease (PD) is the second more common neurodegenerative disorder affecting 1–2% of individuals older than 60 years with an estimated prevalence of 5 million individuals worldwide [1]. PD is primarily characterized by a progressive loss of neuromelanin-containing dopaminergic neurons in the substantia nigra pars compacta (SNc) associated with the appearance of eosinophillic, intracytoplasmic, proteinaceous inclusions termed as Lewy bodies and dystrophic Lewy neurites in surviving neurons [2]. At the time of diagnosis, patients typically display an array of motor impairments including bradykinesia, resting tremor, rigidity, and postural instability. Although most of the typical motor impairments are due to the severe loss of nigrostriatal dopaminergic neurons, PD affects multiple neuronal systems both centrally and peripherally [3], leading to a constellation of non-motor symptoms including olfactory deficits, anxiety and affective disorders, memory impairments, as well as autonomic and digestive dysfunction [4]. These non-motor features of PD, that can appear years, sometimes decades, before the onset of the motor symptoms, do not meaningfully respond to dopaminergic medication and are a challenge to the clinical management of PD [4].

The development of new therapies in PD depends on the existence of representative animal models to facilitate the evaluation of new pharmacological agents and therapeutic strategies before being applied in clinical trials. To date, most studies performed with animal models of PD have focused on their ability to induce nigrostriatal dopaminergic pathway damage and motor alterations associated with advanced phases of PD (for recent review see [5]). As highlighted by Taylor and colleagues [6], since PD is accompanied by alterations of a variety of functions, including olfactory dysfunction [7], [8], anxiety [9], depression [10] and memory deficits [11]–[13], animal models of PD should also display these non-motor behavioral features of this disease.

In this context, some preclinical studies have begun to unravel that the use of low doses and/or particular routes of administration (e.g., intranigral, intrastriatal, intranasal) of some toxins widely used to induce experimental parkinsonism, such as 1-methyl-4-phenyl-1,2,3,6-tetrahydropyridine (MPTP) and 6-hydroxydopamine (6-OHDA), induce a moderate loss of the nigral dopamine neurons resulting in sensorial, emotional and memory deficits with no major motor impairments [14]–[17]. On the other hand, the discovery of mutations associated with familial forms of PD including α-synuclein, Parkin, DJ-1, ubiquitin C-terminal hydrolase L1 T (UCHL1), PTEN-induced putative kinase 1 (Pink1), and Leucine-rich repeat kinase (LRRK2) has led to the generation of genetic mouse models of Parkinsonism (for review see [18]). In comparison with toxin models, the genetic models are at the early stages of behavioral and pharmacological characterization. Therefore, the phenotypical characterization of non-motor symptoms in genetic mouse models of PD constitutes an emerging area of research.

Mutations in *parkin* were first identified as a genetic cause of autosomal recessive juvenile Parkinsonism in Japanese families [19]. More than 100 mutations of the *parkin* gene have been reported, accounting for 50% of familial PD cases and at least 20% of young onset sporadic PD [20]. Parkin functions as an ubiquitin protein ligase with multiple substrates [21], [22]. Although Parkin dysfunction plays a critical role in the general pathogenesis of early onset Parkinsonism, it may also play a role in sporadic PD [18]. Parkin is inactivated by dopaminergic, oxidative and nitrosative stress, which play key roles in sporadic PD [23], [24]. Parkin knockout (^−/−^) mice display impaired ubiquitination and degradation of synaptic vesicle associated proteins [22], mitochondrial dysfunction and increased sensitivity to oxidative stress in dopaminergic neurons [25]. Although there is no evidence for a reduction of nigrostriatal dopamine neurons in Parkin mutant mice, the levels of both dopamine transporter (DAT) and vesicular monoamine transporter (VMAT2) are significantly reduced [26]. Parkin has been suggested to function as a multipurpose neuroprotective agent against a variety of toxic insults, including mitochondrial poisons [27], and is thought to be critical for survival of dopaminergic neurons in PINK1 deficient mice [28]. Accordingly, the viral overexpression of Parkin reduces the MPTP-induced nigral dopamine depletion [29]. However, previous studies failed to show increased vulnerability of *parkin* ^−/−^ mice to dopaminergic neurotoxins such as MPTP [25], [26], [30].

Importantly, two previous studies have used *parkin* ^−/−^ mice to investigate a putative role of Parkin in non-motor behavior [31], [32]. Zhu et al. [31] demonstrated that *parkin* ^−/−^ mice display impaired habituation to a new environment and exhibit increased thigmotaxic behavior and anxiety-related parameters in the light/dark test that may reflect anxiety disorders in PD. *Parkin* null mutant mice also exhibit mild cognitive deficits in the Morris water maze, as indicated by longer escape latencies and failure to selectively cross into the escape platform zone [31]. Moreover, Kurtenback et al. [32] investigated the olfactory function in three genetic PD mouse models and reported that homozygous *parkin* exon 3^−/−^ mice do not display significant alterations in electro-olfactogram recordings (EOGs) and the performance of an olfactory test (cookie-finding test).

Since the establishment of animal models amenable for testing novel therapies to manage the early non-motor symptoms of PD requires a broad behavioral characterization, we now employed adult *parkin* ^−/−^ mice to define the relevance of Parkin in the olfactory, emotional, cognitive and motor functions and in hippocampal synaptic plasticity.

## Materials and Methods

### Ethics Statement

All studies were conducted in accordance with the principles and procedures outlined as “3Rs” in the guidelines of EU (86/609/EEC), FELASA, and the National Centre for the 3Rs (the ARRIVE), and were approved by the Animal Care Committee of the Center for Neuroscience and Cell Biology of Coimbra. We also applied the principles of the ARRIVE guideline for the design and the execution of the in vitro pharmacological experiments (see below) as well as for data management and interpretation and all efforts were made to minimize the number of animals used and their suffering.

### Animals

Experiments were conducted using male homozygous C57BL/6 mice with *parkin* gene deletion on exon 3 (*parkin* ^−/−^) [26] and strain-matched controls (wild-type) with 5–6 months old weighing 25–35 g. A total number of 60 mice were used (30 *parkin* ^−/−^ and 30 littermates) Mice were kept in groups of 4–5 per cage, maintained in a room under controlled temperature (23+1°C) and subjected to a 12-h light/dark cycle (lights on 7:00 a.m.) with free access to food and water. All mice were experimentally naive, and three separate groups of mice were used: group I for olfactory discrimination, forced swimming and rotarod tests; group II for elevated plus-maze, object location, modified Y-maze and biochemical assays of evoked dopamine and glutamate release; and group III for tail suspension, open field habituation, grasping and pole tests and electrophysiological studies. All behavioral, neurochemical and electrophysiological studies were performed by investigators blind to the mouse genotypes.

### Behavioral Tests

All tests were carried out between 9:00 and 14:00 h and they were scored by the same rater in an observation sound-attenuated room under low-intensity light (12 lx), where the mice had been habituated for at least 1 h before the beginning of the tests. Behavior was monitored through a video camera positioned above the apparatuses and the videos were later analyzed with the ANY Maze video tracking system (Stoelting Co., Wood Dale, IL, USA). The apparatus were cleaned with 10% ethanol between animals to avoid odor cues.

### Olfactory Discrimination

The olfactory discrimination ability of mice was assessed with an olfactory discrimination test that we have previously used [15]. The task takes advantage of the fact that rodents prefer places impregnated with their own odor (familiar compartments) instead of places with non-familiar odors. Briefly, each mouse was placed for 5 min in a cage divided in two equal areas separated by an open door, where it could choose between one compartment with fresh sawdust (non-familiar compartment) and another with unchanged sawdust (familiar compartment) that the same mouse had occupied for three days before the test. The following parameters were registered: time (s) spent in each compartment (familiar *versus* non-familiar) and the number of crossing between the two compartments.

### Elevated Plus-Maze

The elevated plus-maze was used on the basis of its documented ability to detect both anxiolytic- and anxiogenic-like drug effects in mice [33]. Briefly, the apparatus consisted of four arms were 18 cm long and 6 cm wide that were made of wood covered with impermeable formica, and placed 60 cm above the floor. Two opposite arms were surrounded by walls (6 cm high, closed arms), while the other two were devoid of enclosing walls (open arms). The four arms were connected by a central platform (6×6 cm). Each mouse was placed in the center of the maze facing a closed arm and their behavior was monitored for 5-min: anxiogenic-like effects were defined as a decrease in the proportion of open arm entries divided by the total number of arm entries, and the time spent on open arms relative to the total time spent on both arms. Whenever a mouse placed all four paws onto an arm, an entry was recorded. The total number of closed arm entries was utilized as a measure of locomotor activity.

### Tail Suspension

The tail suspension test has become one of the most widely used tests for assessing antidepressant-like activity in mice. It is based on the fact that animals subjected to the short-term inescapable stress of being suspended by their tail, will develop an immobile posture. The total duration of immobility induced by tail suspension test was measured according to the method described by Steru et al. [34]. Briefly, mice were suspended 50 cm above the floor with an adhesive tape placed approximately 1 cm from the tip of their tail. Immobility time was recorded during a 6 min period. Mice were considered immobile only when they hung passively and completely motionless.

### Forced Swimming

The forced swimming test [35] was carried out in mice individually forced to swim in an open cylindrical container (diameter 10 cm, height 25 cm), containing 19 cm of water at 25+1°C. During the 6 min test session, the following behavioral responses were recorded by a trained observer: the immobility time (i.e. the time spent floating in the water without struggling, making only those movements necessary to keep the head above the water) and climbing behavior, which is defined as upward directed movements of the forepaw along the cylinder walls. Decrease in immobility time is indicative of a reduced depressive-related behavior while time of climbing was used as a predictor of altered motor activity scored directly in the forced swimming test [36].

### Accelerating Rotarod

Mice were placed on a rotarod apparatus (Columbus Instruments, USA) accelerating from 4 to 40 rpm in 5 min. Trials began by placing the mouse on the rod and beginning rotation. Each trial ended when the mouse fell off the rod, and latency was recorded. Mice were tested for four trials a day (1-min inter-trial interval) for 3 consecutive days [37].

### Grasping Strength

The grasping strength test was carried out as described previously [38]. A wire grid measuring 8 cm×14 cm (wire diameter: 1.5 mm) was connected to an ordinary electronic scale by four sticks 15 cm long. Mice were allowed to grasp the grid while being held by the tail with increasing firmness until they loosened their grip. The value registered by the scale at the precise moment of loosening was noted as the grasping strength (g). Mice were tested three times and the best value of performance was recorded. To avoid false measurements due to wrist flexion, the situation of four digits grasping in the center of the grid was the only one accepted [38].

### Pole

The pole test assesses the agility of animals and may be a measure of bradykinesia. A vertical wooden pole with a rough surface (50 cm in height and 1 cm in diameter) was placed in the home cage. Mice placed head-up on top of the pole, orient themselves downward and descend the pole back into their home cage. On the test day, animals were exposed to five trials, and the time spent to orient downward (t-turn) and the time to descend (t-descend) were measured. The best performance over five trials was used. If the mouse was unable to turn completely downward, fell or slipped down, the default value of 120 s was taken as maximal severity of impairment [39].

### Open Field Habituation

Mice were placed into the center of the square arena (50 cm wide ×50 cm deep ×40 cm high) made of grey PVC for 60 min on two consecutive days. The distance traveled (m) was collected in 5-min intervals [40].

### Object Location

The spatial memory of mice was assessed with the object location task, which has been used to study hippocampal-dependent memory [41]. The task is based on the spontaneous tendency of rodents, previously exposed to two identical objects, to later explore one of the objects (replaced in a novel location) for a longer time than they explore the non-displaced object, and has been used for the evaluation of hippocampal-dependent memories [41]. The apparatus used was an open-field box (50 cm wide ×50 cm deep ×40 cm high) made of grey PVC. Identical plastic rectangles (4 cm high ×4.5 cm wide) were used as objects. The protocol used was based on the previously described by Assini et al. [41]. The mice were placed in the center of the apparatus with two identical objects for 5 min. The objects were placed 7 cm away from the walls of the open field. The exploration of the objects was recorded by a stopwatch when mice sniffed, whisked, or looked at the objects from no more than 1 cm away. After the training phase, the mice were removed from the apparatus for 180 min. After the inter-trial interval, one object was moved to a new location. The time spent by the animals exploring the objects in new (novel) and old (familiar) locations was recorded during 5 min. All locations of the objects were counterbalanced among the groups. In order to analyze the cognitive performance, a location index was calculated as previously described [42]: (T_novel_×100)/(T_novel_+T_familiar_), where T_novel_ is the time spent exploring the displaced object and T_familiar_ is the time spent exploring the non-displaced object.

### Modified Y-Maze

The modified Y-maze task was used to assess short-term spatial memory and is based on the innate preference of animals to explore areas that have not been previously explored [43]. The Y-maze apparatus consisted of three arms (18 cm long, 6 cm wide and 6 cm high) made of wood covered with impermeable Formica elevated to a height of 50 cm above the floor. This task consisted of two trials (training and test) of 8 min each separated by an inter-trial interval of 120 min. During the training trial, one arm (“novel”) was blocked by a removable door and the mice were placed at the end of the one arm (“start”) facing the center and they could chose between the start and the “other” arm. At the end of the training trial, the mouse was removed from the maze and kept in an individual cage during the inter-trial interval (120 min). During the test trial, the “novel” arm was opened and the animals were once again placed at the start arm and allowed to explore freely the three arms during 8 min. The number of entries and the time spent in each arm were recorded. Entry into an arm was defined as placement of all four paws into the arm.

### Extracellular Hippocampal Electrophysiology

Electrophysiological recordings were carried out as previously described [44], [45]. Briefly, mice (wild-type and *parkin* ^−/−^) were deeply anesthetized under a halothane-saturated atmosphere (Sigma-Aldrich, St Louis, MO, USA) before decapitation. Brains were quickly removed and placed in ice-cold standard artificial cerebrospinal fluid (aCSF) containing (in mM); 124 NaCl, 4.5 KCl, 2 CaCl_2_, 1 MgCl_2_, 26 NaHCO_3_, 1.2 NaH_2_PO_4_ and 10 D-glucose, gassed with a gas mixture of 95% O_2_ and 5% CO_2_. The hippocampi were cut in 400 µm thick transverse slices using a McIlwain tissue chopper (Mickle Lab Engineering, Guildford, UK) and kept in oxygenated aCSF at room temperature for at least 60 min, before being used. Individual slices were transferred to a recording chamber and superfused with oxygenated aCSF at 30.5°C at a flow rate of 3 mL/min. Bipolar stainless steel electrodes were placed on the Shaffer collateral/comissural fibers and test stimuli were delivered via a S44 stimulator (Grass Instruments, West Warwick, RI) with a stimulus isolation unit (PSIU6, Grass Instruments) at a frequency of 0.06 Hz. Glass microelectrodes (1–2 MΩ) backfilled with 4 M NaCl were used to record field excitatory postsynaptic potentials (fEPSPs) in the stratum radiatum of the CA1 region of hippocampus. Recordings were obtained using an ISO-80 amplifier (World Precision Instruments, Hertfordshire, UK) and digitized using an ADC-42 board (Pico Technologies, Pelham, NY, USA). Averages of 4 consecutive responses were continuously monitored on a personal computer with the LTP 1.0.1 software [44].

An input-output curve was first carried out to evaluate the threshold to the maximum response and the working stimulus intensity was adjusted to evoke fEPSPs of half maximal amplitude (50%). Short-term plasticity was gauged using a paired-pulse facilitation (PPF protocol) consisting of two identical stimuli separated by an inter-stimulus interval of 25, 50, 100, 200 and 400 ms and the ratio between the second and the first fEPSP was calculated. We also used a theta burst protocol to evaluate long-term potentiation (LTP), which consisted of 10 bursts with four pulses at 100 Hz with 200 ms inter-burst interval; the fEPSPs were recorded for additional 60 min [45]. The average slope of the fEPSP at baseline was set at 100%, and changes of the fEPSP slope were expressed as percent of change from baseline.

### Dual-label [^3^H]Dopamine/[^14^C]Glutamate Release from Striatal Nerve Terminals

Neurotransmitter release experiments were carried out as described before [46], [47] using purified nerve terminals, termed synaptosomes [48], which represent an excellent tool to study presynaptic processes free of polysynaptic and glial influences [49]. Pairs of striata were quickly removed into 2 mL ice-cold sucrose solution (0.32 M, containing 5 mM HEPES, pH 7.4), homogenized instantly with a Teflon homogenizer, and centrifuged at 5,000 rpm for 5 min. The supernatant was centrifuged at 13,000 rpm for 10 min to obtain the P2 crude synaptosomal fraction as a pellet. Synaptosomes were then diluted to 0.5 mL with Krebs' solution (in mM: NaCl 113, KCl 3, KH_2_PO_4_ 1.2, MgSO_4_ 1.2, CaCl_2_ 2.5, NaHCO_3_ 25, glucose 10, HEPES 15, pH 7.4, 37°C), containing the MAO-B inhibitor, pargyline (10 µM, Sigma) to prevent [^3^H]dopamine degradation, and the glutamate decarboxylase inhibitor, aminooxyacetic acid (100 µM, Sigma) to prevent [^14^C]glutamate metabolism. Under these conditions, synaptosomes in pair (obtained from one wild-type and one *parkin* ^−/−^) were incubated with [7,8-^3^H(N)]dopamine ([^3^H]dopamine; final concentration: 200 nM; American Radiolabeled Chemicals, Saint Louis, MO, USA) and L-[^14^C(U)]glutamic acid ([^14^C]glutamate; final concentration: 20 µM; American Radiolabeled Chemicals), in a final volume of 250 µL at 37°C, for 10 min. Synaptosomes then were transferred to four microvolume chambers (∼0.17 mg protein/130 µL/chamber) - *i.e.* experiments ran in quadruplicate, and were trapped in Whatman GF/C filters (Sigma), and superfused (washed) with Krebs' solution at 37°C for 10 min, at a rate of 0.8 mL/min.

After collecting four 2-min samples as baseline, the evoked release of the transmitters was stimulated twice with KCl: S1, first stimulus, 20 mM KCl and S2, 75 mM KCl (both isomolar substitution of NaCl), each for 1 min, with a 10-min interval. We used 20 mM KCl as a weak and 75 mM KCl as a strong stimulus in order to compare the two animal strains under different stimulus paradigms. In the wild-type mice, repetitive stimulations of the same synaptosomal preparations with 75 mM KCl triggered similar responses (data not shown), indicating that short 75 mM KCl pulses do not prejudice synaptosomal functionality. When the Ca^2+^-dependence of the resting and the evoked release was tested, Ca^2+^ concentration was diminished to 100 nM, and MgCl_2_ was elevated to 10 mM in the Krebs' solution to block Na^+^ entry through voltage-gated Ca^2+^ channels [50], which would otherwise cause the reversal of the membrane transporters.

After the experiments, the radioactivity content of each samples and the filters with the trapped synaptosomes were counted by a single or a dual-label protocol using a Tricarb β-counter (PerkinElmer), and DPM values were expressed as fractional release (FR%), i.e. the percent of actual content in the effluent as a function of the total synaptosomal content.

### Statistical Analysis

All the data were tested for normality by the Kolmogorov-Smirnov normality test. Except for the dopamine and glutamate release experiments, the values are expressed as means ±S.E.M. (n equals the number of mice included in each analysis). Differences between wild type and *parkin* ^−/−^ groups were analyzed using unpaired Student's *t*-test or one-way analysis of variance (ANOVA) with repeated measures (trials). The % of change from electrophysiological experiments represents the quantification of the last 10 min of the LTP process. The accepted level of significance for the tests was P≤0.05. Results from the release experiments are means ±S.E.M. of 7 pairs of animals in quadruplicate. Stimulation-induced transmitter release values (S1 and S2 values) were calculated by the area-under-the curve protocol [50]. Basal release values were determined as the mean of the first three data points of the release curve before S1. Uptake was determined in a batch protocol during 10 min, in paired condition and expressed to the normocalcemic results of the same animal, and was compared with the help of paired *t*-test. Low-Ca^2+^ and *parkin* ^−/−^ data (basal, S1, S2, S2/S1, and uptake values) were normalized (i.e. expressed as % of the respective wild-type values from the same paired experiment), and statistically analyzed by the one-sample *t*-test against the hypothetical value of 100 (%). P<0.05 was accepted as significant difference. All tests were performed using GraphPad Prism 5.0 software package.

## Results

### Genetic Deletion of *Parkin* Impairs Open Field Habituation

**Figure 1:**
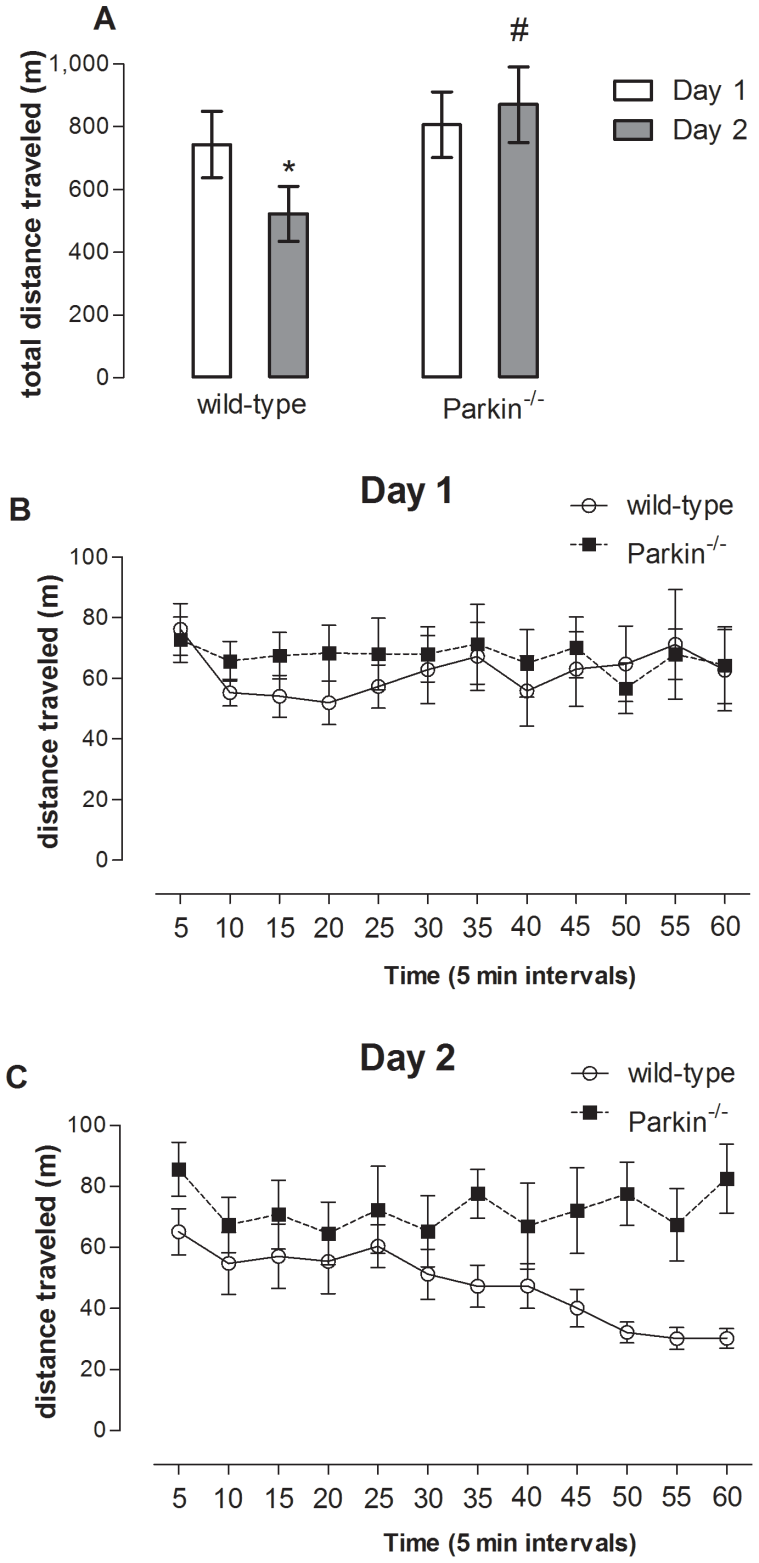
Effects of *parkin* genetic deletion on locomotor activity and habituation. (A) total distance traveled by wild-type and *parkin* ^−/−^ mice at days 1 and 2 of analysis in an open field arena. *P<0.05 compared to day 1, *^#^P*<0.05 compared to day 2 of wild-type group using a Newman-Keuls post-hoc test. (B) Pattern of locomotion at day 1 (divided in blocks of 5 min) of wild-type and *parkin* ^−/−^ mice. (C) Pattern of locomotion at day 2 (divided in blocks of 5 min) of wild-type and *parkin* ^−/−^ mice. Data are mean ± s.e.m. of n = 9–10 per group.

The locomotor activity of wild-type and *parkin* ^−/−^ was assessed in the open field test. Our results indicate similar locomotor activity in the first exposition to the open field between wild-type and *parkin* ^−/−^ mice (genotype factor F_(1,36)_ = 1.88, *P>0.05)*, with the same distribution over the period of analysis (repetition factor F_(1,36)_ = 0.02 *P>0.05*), and interaction between the factors (genotype *vs.* repetition F_(1,36)_ = 0.59 *P>0.05)* (Fig. 1a). However, the results from the second exposition indicated that the wild-type mice displayed an habituation response (i.e., decreasing the exploration of the apparatus), while *parkin* ^−/−^ mice spent the same amount of time investigating the apparatus (Fig. 1a) (genotype factor F_(1,36)_ = 1.90 *P>0.05)* (Fig. 1b)*;* (repetition factor: F_(1,36)_ = 3.02; *P*<*0.05*) and (genotype *vs*. repetition factor (F_(1,36)_ = 5.40; *P*<*0.05*) (Fig. 1c).

### Genetic Deletion of *Parkin* Disrupts Hippocampal-Dependent Memory in Mice

**Figure 2:**
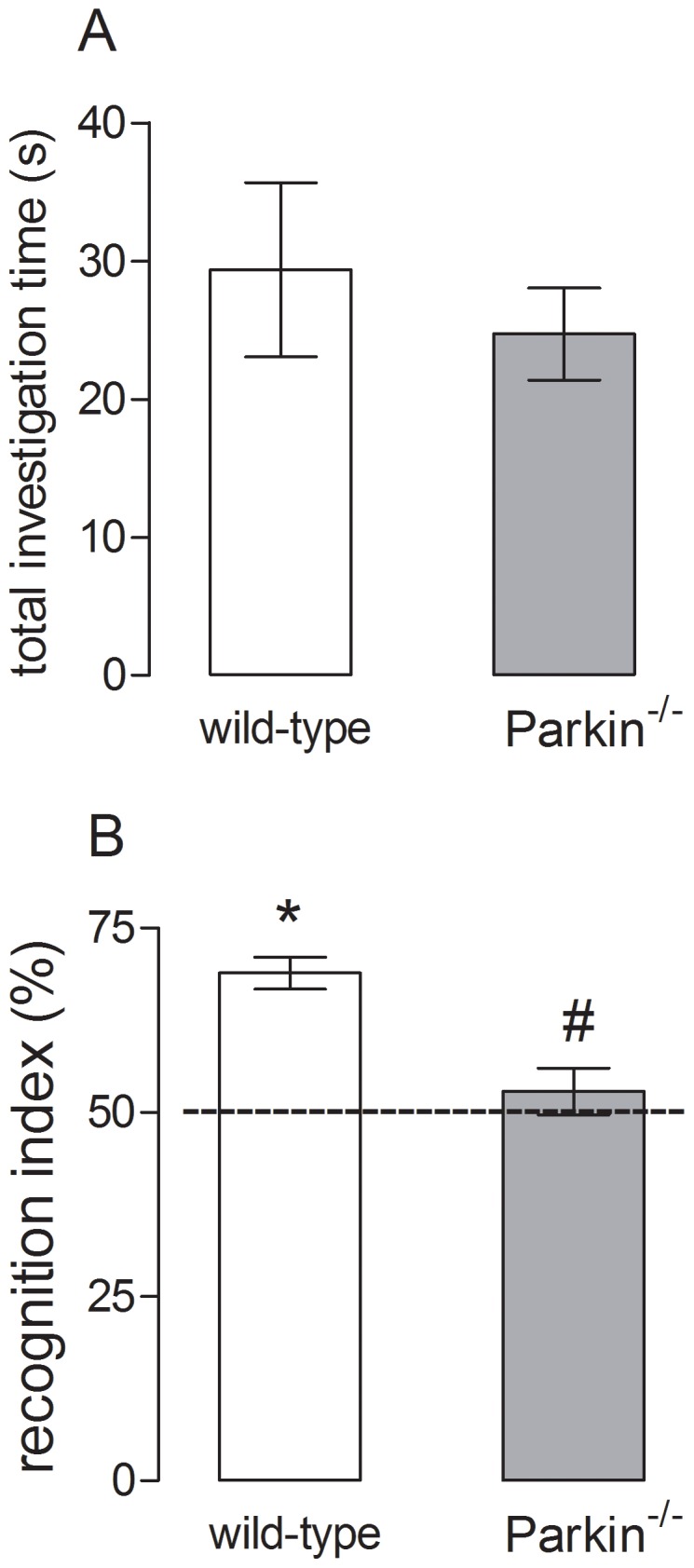
Effects of *parkin* genetic deletion on the spatial memory performance. (A) total investigation time by wild-type and *parkin* ^−/−^ mice during the training session. (B) Recognition index of wild-type and *parkin* ^−/−^ mice during the test session. *P<0.05 compared to the hypothetical 50% (random investigation). *^#^P*<0.05 compared to the wild-type control group. Data are mean ± s.e.m. of n = 9–10 per group.

The effects of the genetic deletion of *parkin* on learning and memory were evaluated using the object location and the modified Y-maze tasks. The results from object location task indicated that wild-type and *parkin* ^−/−^ mice spent similar time investigating the objects (t_2,0.05_ = 0.63, *P>0.05*) (Fig. 2a), but *parkin* ^−/−^ displayed a lower recognition index (t_2,0.05_ = 4.27, *P*<*0.05*) in in comparison to wild-type mice (Fig. 2b).

**Figure 3:**
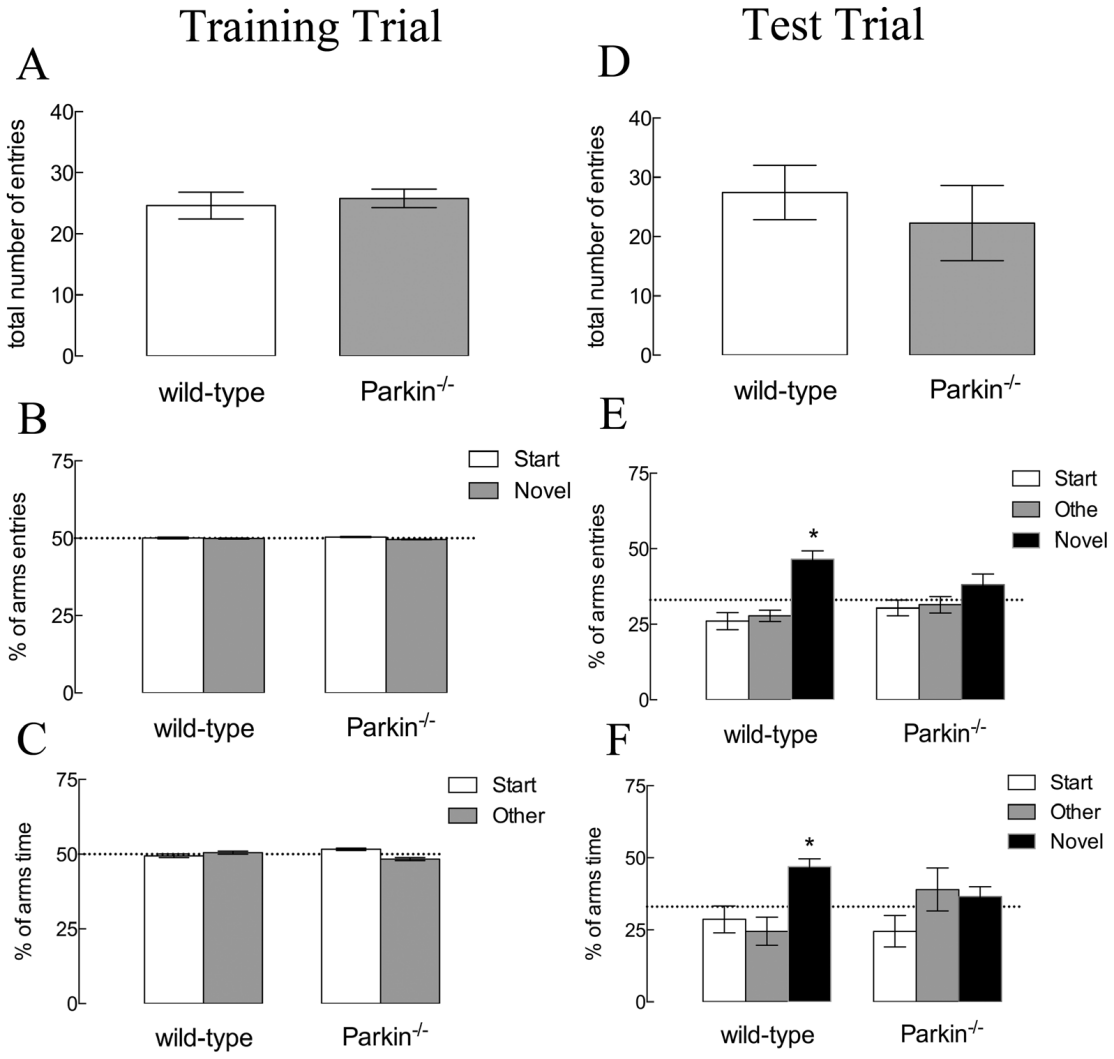
Effects of *parkin* genetic deletion on spatial recognition memory performance. (A and D) Total number of entries during the training and the test sessions (respectively) by wild-type and *parkin* ^−/−^ mice. (B and E) Percentage of arms' entries during the training and test sessions (respectively); *P<0.05 compared to the hypothetical value of 33% (random entries). (C and F) Percentage of time spent in each arm during the training and test sessions (respectively) by wild-type and *parkin* ^−/−^ mice; *P<0.05 compared to the hypothetical value of 33% (random time). Data are mean ± s.e.m. of n = 9–10 per group.

The statistical analysis of the training trial of the modified Y-maze indicated similar number of total entries (t_2,0.05_ = −0.42, *P>0.05*) (Fig. 3a), % of entries in the starting arm (t_2,0.05_ = −0.27, *P>0.05*), % entries in the other arm (t_2,0.05_ = 0.27, *P>0.05*) (Fig. 3b) and also similar % of time in the starting arm (t_2,0.05_ = −0.41, *P>0.05*) and % of time in the other arm (t_2,0.05_ = 0.41, *P>0.05*) (Fig. 3c). The statistical analysis of the test trial of the modified Y-maze revealed once again similar number of entries in the arms (t_2,0.05_ = 1.65, *P>0.05*) (Fig. 3d). However, despite same % of entries in the starting arm (t_2,0.05_ = −1.11, *P>0.05*) and % of entries in the other arm (t_2,0.05_ = −1.13, *P>0.05*) *parkin* ^−/−^ mice presented decreased % of entries in the novel arm (t_2,0.05_ = 2.90, *P*<*0.05*) (Fig. 3e) when compared to wild-type mice. Likewise, the % of time spent in the starting arm and the % of time spent in the other arm were similar between the groups (t_2,0.05_ = 0.80, *P>0.05;* and t_2,0.05_ = −1.69, *P>0.05,* respectively) but the % of time spent in the novel arm was decreased in *parkin* ^−/−^ mice (t_2,0.05_ = 3.89, *P*<*0.05*) when compared to wild-type mice (Fig. 3f).

### Genetic Deletion of *Parkin* Decreases Long-Term Potentiation in The Hippocampus

**Figure 4:**
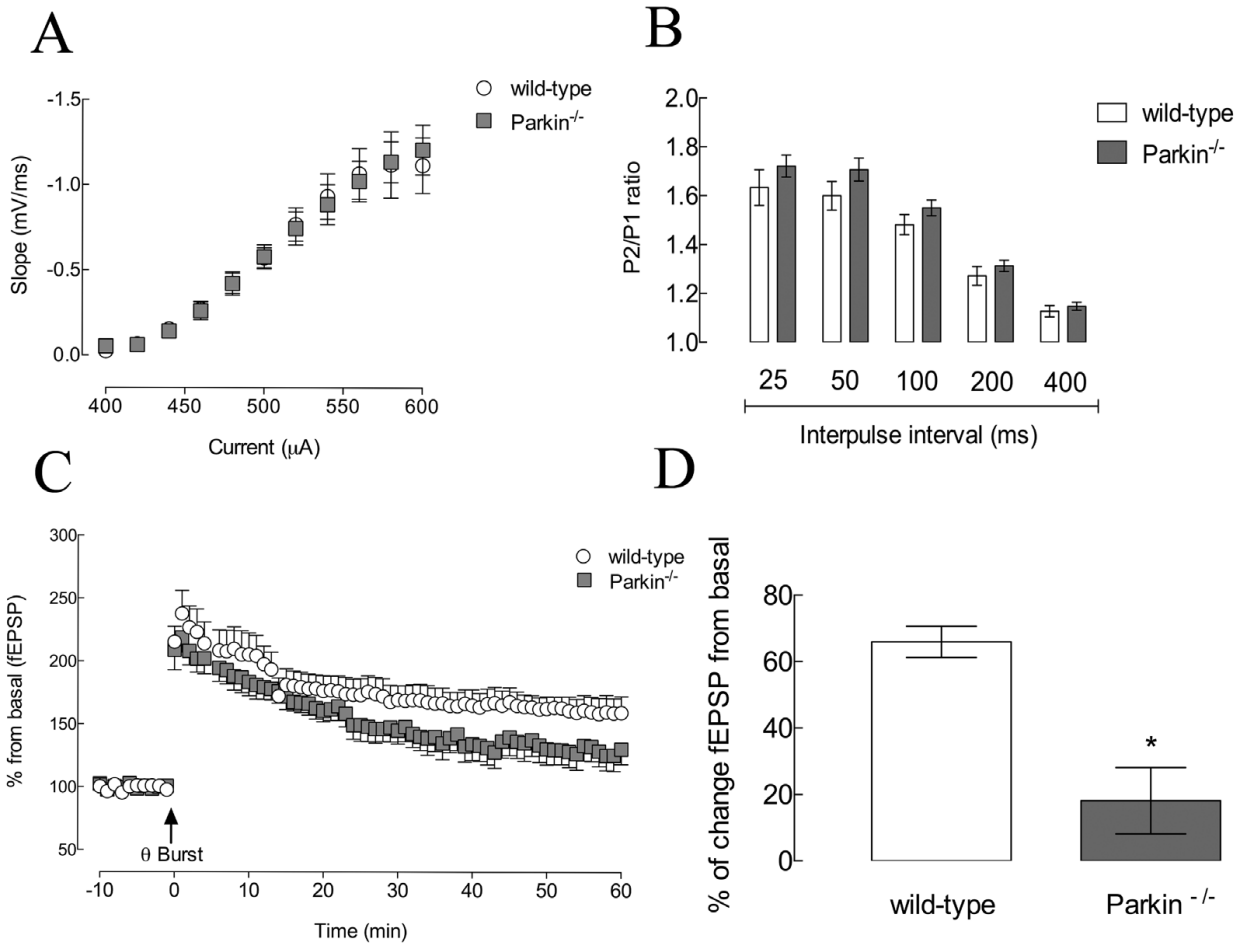
Effects of *parkin* genetic deletion on hippocampal synaptic transmission and plasticity processes. (A) Input-output curve of field excitatory post-synaptic potentials (fEPSP, measured as their slope) recorded in Schaffer fibers-CA1 pyramid synapses of hippocampal slices from wild-type and *parkin* ^−/−^ mice. (B) Paired pulse facilitation (PPF), measured as the ratio of fEPSPs' slope ratio between the second and first paired stimuli (P2/P1 ratio) with different interpulse intervals (25, 50, 100, 200 and 400 ms) recorded in hippocampal slices from wild-type and *parkin* ^−/−^ mice. (C) Modification of fEPSPs' slope, presented as % of baseline, before and after θ-burst stimulation in slices from wild-type and *parkin* ^−/−^ mice. (D) Potentiation of fEPSPs' slope after the θ-burst stimulation, presentage as % increase of baseline fEPSPs' slope, in slices from wild-type and *parkin* ^−/−^ mice *P<0.05 compared to wild-type control group. Data are mean ± s.e.m. of n = 4 per group.

Hippocampal electrophysiology in slices from wild-type and *parkin* ^−/−^ mice revealed the same synaptic density as shown by the similar input-output curve in the two groups of mice (t_2,0.05_ = 0.14, *P>0.05*) (Fig. 4a). Paired-pulse stimulation protocol also yielded similar results in slices from wild-type and *parkin* ^−/−^ mice for all the inter-pulse intervals (*P>0.05*) (Fig. 4b). However, we observed a decrease of the amplitude of the Θ-burst stimulation-induced long-term potentiation in slices from *parkin* ^−/−^ mice when compared to slices from wild-type mice (t_2,0.05_ = 4.59, *P*<*0.05*) (Fig.s 4c and d).

### The *Parkin* ^−/−^ Mice Leak Dopamine from the Nerve Terminals

**Figure 5:**
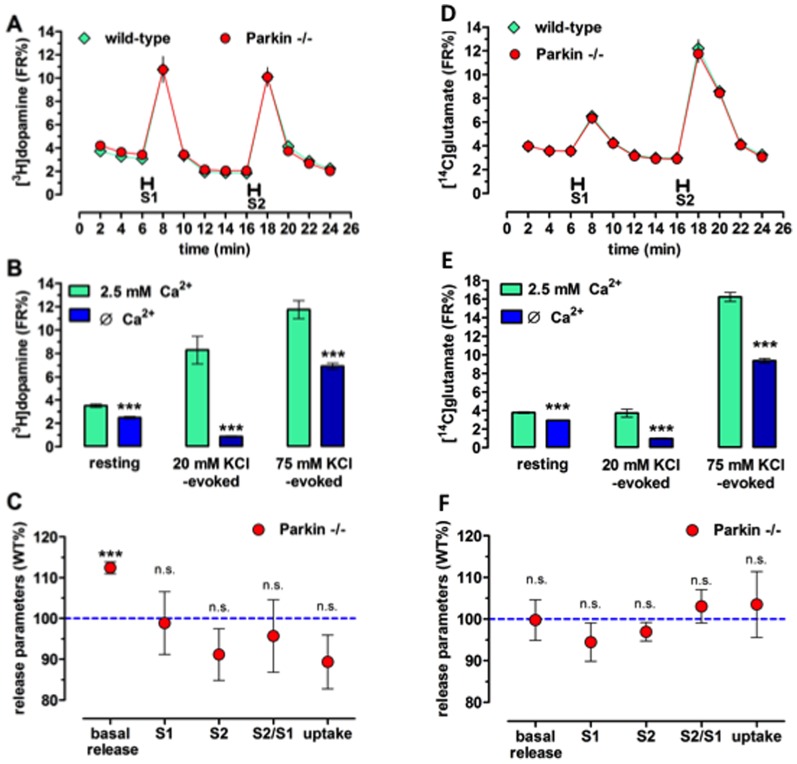
Basal rather than stimulated [^3^H]dopamine release is increased in striatal synaptosomes of parkin^−/−^ *versus* wild-type mice, whereas [^14^C]glutamate release is unchanged. Time course of the averaged time course of [^3^H]dopamine release (A) and [^14^C]glutamate release (D) from wild-type and parkin^−/−^ mice. S1, S2: stimulation for 1 min with 20 and 75 mM KCl. Ca^2+^-dependence of the resting and stimulated release of [^3^H]dopamine (B) and [^14^C]glutamate (E). Low-calcium experiments were carried out in the presence of 100 nM CaCl_2_ and 10 mM MgCl_2_. Release and uptake parameters of [^3^H]dopamine (C) and [^14^C]glutamate (F) from parkin^−/−^ mice normalized to the wild-type mice in the pairwise experiments. ***p<0.001, calculated with paired t-test (B,E) and one-sample t-test (C). Data are mean ± s.e.m. of n≥7 per group with each experiment performed in quadruplicate.

To test whether Parkin deficiency alters the presynaptic release of two neurotransmitters with pivotal role in PD, i.e. dopamine and glutamate, we compared basic release parameters in wild-type *versus parkin* ^−/−^ mice a single layer of vertically superfused synaptosomes, where there is no interaction among the seeded nerve terminals and their neurotransmitters, thus allowing to study pure presynaptic release parameters [49]. We also aimed at testing this ability of the nerve terminals to take up (uptake level) and release the neurotransmitters without stimuli (basal release, representing spontaneous activity), or upon a low stimulus (20 mM KCl) and a high stimulus (75 mM KCl). The resting and the evoked releases of the two transmitters were Ca^2+^-dependent (Figs. 5B, E).

Under this paradigm, neither the uptake nor the stimulus-evoked release of [^3^H]dopamine was significantly (*P*>0.05) affected by the deletion of the *parkin* gene (Figs. 5A, C). In contrast, the basal release of dopamine was significantly higher in the *parkin* ^−/−^ mice (t_2,0.05_ = 8.58, *P*<*0.05*), which probably represents transporter-mediated leakage since the Ca^2+^-dependent (vesicular) release was not statistically different between the strains (*P>0.05*, Figs. 5A, C).

As for glutamate, neither the uptake nor the basal or stimulus-evoked release values were significantly different between the two strains (Figs. 5D, F).

## Discussion

The importance of *parkin* for the pathogenesis of PD has been well established [20]. Here we hypothesized that Parkin could also be involved in the emergence of the premotor symptoms of PD. Our study provides new evidence that the genetic deletion of *parkin* causes a short-term memory decline (observed during the premotor stage of PD) corroborated by modifications in the hippocampal synaptic plasticity.

The genetic deletion of *parkin* did not modify the olfactory ability, locomotor activity (in the first exposure to the open field) (Fig. 1a), and emotional responses (namely anxiety-like and depressive-like behaviors) in mice (Table 1). The abovementioned result prompts the suggestion that *parkin* might not be involved in all premotor symptoms but it is selectively important to control memory performance.

**Table 1:** Summary of the performance of wild-type and *parkin* knockout (*parkin* ^−/−^) mice on behavioral tests evaluating olfactory, emotional and motor functions.

Behavioral test	Parameter	Subjects	
		wild-type	*parkin* ^−/−^
**Olfactory discrimination**	Familiar compartment time (%) Non-familiar compartment time (%) Number of crossings	62.8±2.9 n = 10 37.2±2.9 12.1±1.2	58.5±3.6 n = 9 41.5±3.6 15.8±1.2
**Elevated plus-maze**	Open arms time (%) Open arms entries (%) Number of closed arms entries	4.0±1.6 n = 11 11.7±5.1 9.5±1.1	6.3±1.4 n = 10 13.8±2.7 13.2±0.8
**Forced swimming**	Immobility time (s) Climbing time (s)	133.5±17.7 n = 10 51.7±8.2	162.4±13.7 n = 9 53.2±5.6
**Tail suspension**	Immobility time (s)	154.4±15.6 n = 11	166.8±7.6 n = 10
**Rotarod**	Latency to fall (s) – Day Latency to fall (s) – Day 2 Latency to fall (s) – Day 3	73.1±4.9 n = 10 90.2±2.2 99.9±4.1	66.8±5.8 n = 9 92.5±5.9 104.9±7.7
**Grasping strength**	Grasping strength (g)	51.6±3.2 n = 11	53.8±3.5 n = 10
**Pole**	Turn latency (s) Descent latency (s)	2.3±0.3 n = 11 5.0±0.4	2.8±0.5 n = 10 4.5±0.3

Data are expressed as mean ± s.e.m. of 9–11 animals per group. Statistical analysis revealed absence of significant differences between wild-type and *parkin* ^−/−^ mice in the performance of such behavioral test.

The first clue for possible differences in the cognitive ability was observed in the open field habituation test. During the second exposition of wild-type and *parkin* ^−/−^ mice to the open field arena, *parkin* ^−/−^ mice displayed a lack of novelty habituation. This type of habituation is normally related to a modified hippocampal function, since the hippocampus is important for the recognition of a new environment and for the decreased locomotor activity in an environment that was already explored and recognized [51]. Remarkably, patients carrying one mutation of the *parkin* gene display a cognitive decline (in the face recognition test) in a premotor situation when compared to control subjects [52].

Following the possibility that the lack of Parkin might affect information processing in hippocampal circuits, we performed the object location task, which is critically dependent on the CA1 region of the hippocampus [41]. Our results showed that the genetic deletion of *parkin* decreased the recognition index without interfering with the total investigation time. The genetic deletion of *parkin* also induced a cognitive decline in the modified Y-maze task, which measures spatial recognition memory [53], [54] depending on the integrity of hippocampal circuits [55] and NMDA receptors in the CA1 area [56] and plastic changes in hippocampal circuits [57]. The decrease in memory performance observed here was also described in other animal models of PD, like in the MitoPark mice [58] and after the intranasal administration of MPTP [59], reinforcing the cognitive aspect of PD and the importance of Parkin in this context. Combining the results from our behavioral evaluation and from other studies, the *parkin* ^−/−^ mice present an early PD phenotype, with olfactory and cognitive deficits (both characteristic findings of early-PD), but lacking locomotor alterations [31], [32]. Additionally, two studies on the mitochondrial profile in *parkin* ^−/−^ mice also suggested that Parkin could be a marker of early PD [60], [61]. It is important to mention that our behavioral analysis is complimentary with two previous works [31], [32] differing only in the emotional evaluation. Zhu and colleagues found an anxiogenic profile in *parkin* ^−/−^ mice not found in our study. One important difference between the two studies was the test used to evaluate anxiety, while we utilized the elevated plus-maze, Zhu and colleagues utilized the light-dark box test, which uses a more intense aversive stimulus, and probably allows the observation of more discrete differences between the groups.

Subsequently, we electrophysiologically evaluated alterations of synaptic transmission and plasticity in *parkin* ^−/−^ mice focusing on hippocampal Schaffer fiber-CA1 pyramid synapses. We did not observed differences in the input-output curve indicating a preservation of synaptic density in *parkin* ^−/−^ mice. Furthermore, short-term plasticity using a PPF protocol, which is mostly dependent on pre-synaptic calcium handling [62], was unaltered in *parkin* ^−/−^ mice, suggesting a preserved pre-synaptic function in *parkin* ^−/−^ mice; however, Θ-burst stimulation triggered a long-term potentiation with lower amplitude in slices from *parkin* ^−/−^ mice compared to wild-type mice. Since synaptic plasticity is considered a neurophysiological basis of memory processing [63], this observed decrease of Θ-burst-induced LTP amplitude in hippocampal slices from *parkin* ^−/−^ mice, further corroborates our behavioral results showing deficits of hippocampal-dependent memory performance in *parkin* ^−/−^ mice. Synaptic plasticity processes are critically dependent on the engagement NMDA receptors and it is worth noting that it was previously shown that Parkin interacts with proteins contained in the post-synaptic PDZ domain including the NMDA receptor [64]. This prompts the possibility that the absence of Parkin could lead to a destabilization of post-synaptic NMDA receptors, leading to deficits of synaptic plasticity resulting in cognitive dysfunction.

Previous studies in *parkin* ^−/−^ mice reported different alterations of hippocampal synaptic plasticity, which were only observed in aged mice [65]. However it is important to mention fundamental differences in the electrophysiological protocols: 1) in the abovementioned study the authors used picrotoxin (an antagonist of GABA_A_ receptors) to avoid population spikes contaminations, a common approach when aging animals are utilized but that can also influence the amplitude of LTP [66]; 2) Hanson et al. also used a different protocol to induce the LTP, namely high frequency stimulation, whereas we used the Θ-burst stimulation because it mimics in vivo firing frequencies (3–12 Hz) in the CA1 region of rodents performing a spatial learning task, being recognized as more similar to the physiological stimulation of the CA1 area [67], [68]. Still, it is argued that the Θ-burst stimulation is more efficient in the induction of LTP in comparison to HFS protocol [69].

We also studied the basal and stimulated release of a neurotransmitter (glutamate) and neuromodulator (dopamine) in the striatum, the signaling molecules and brain area typically affected in PD. The stimulation with 20 mM or 75 mM K^+^ evoked similar Ca^2+^-dependent release of glutamate and dopamine from synaptosomes of wild-type and the *parkin* ^−/−^ mice. Furthermore, no change in the S1/S2 amplitude-ratio was observed, which is in agreement with the lack of alterations of PPF in the hippocampus. However, striatal synaptosomes from *parkin* ^−/−^ mice displayed a higher basal release of dopamine, but not of glutamate, thus ruling out energy shortage of compromised viability as a possible cause for this difference. The basal release also exhibited significant Ca^2+^-dependency – the so-called spontaneous vesicular release fraction –, although the majority of basal release is Ca^2+^-independent, and hence, transporter-dependent, thus of cytosolic origin [62]. Thus, the most parcimonous interpretation is that the *parkin* ^−/−^ mice have increased cytoplasmic dopamine level as a result of decreased vesicular monoamine transporter activity or a greater cytoplasmic dopamine outflow due to increased dopamine transporter density.

In summary, the present study provides evidence indicating that the genetic deletion of *parkin* alters the short-term spatial memory and hippocampal synaptic plasticity in mice. Taken together, the results of the present study indicate that Parkin is relevant for the hippocampal functioning. However, further investigation is needed to identify the molecular pathways responsible for these modifications.
