# Correction: Behavioral Phenotyping of Parkin-Deficient Mice: Looking for Early Preclinical Features of Parkinson's Disease

**DOI:** 10.1371/journal.pone.0118526

**Published:** 2015-03-05

**Authors:** The PLOS ONE Staff

The following information is missing from the Funding section: Additionally, this work was supported by Fondation ICM, “Investissements d’avenir” ANR-10-IAIHU-06. The complete, correct funding information is as follows: This work was supported by grants from Conselho Nacional de Desenvolvimento Científico e Tecnológico (CNPq), Coordenação de Aperfeiçoamento de Pessoal de Nível Superior (CAPES-COFECUB 681–10), Programa de Apoio aos Núcleos de Excelência (PRONEX—Project NENASC), Fundação de Apoio à Pesquisa do Estado de Santa Catarina (FAPESC), QREN (CENTRO-07-ST24-FEDER-002006). Additionally, this work was supported by Fondation ICM, “Investissements d’avenir” ANR-10-IAIHU-06. DR and AAC received scholarships from CNPq. RDP is supported by a research fellowship from CNPq. RRV and RAC are supported by Visitant Research fellowships from CNPq (Ciência sem Fronteiras). The authors have no financial or personal conflicts of interest related to this work. The funders had no role in study design, data collection and analysis, decision to publish, or preparation of the manuscript.
